# Study of InN epitaxial films and nanorods grown on GaN template by RF-MOMBE

**DOI:** 10.1186/1556-276X-7-468

**Published:** 2012-08-21

**Authors:** Wei-Chun Chen, Shou-Yi Kuo, Wei-Lin Wang, Jr-Sheng Tian, Woei-Tyng Lin, Fang-I Lai, Li Chang

**Affiliations:** 1 Instrument Technology Research Center, National Applied Research Laboratories, 20 R&D Road V1, Hsinchu Science Park, Hsinchu, Taiwan, 300, Republic of China; 2 Department of Electronic Engineering, Chang Gung University, 259 Wen-Hwa 1st Road, Kwei-Shan, Tao-Yuan, Taiwan, 333, Republic of China; 3 Department of Materials Science and Engineering, National Chiao Tung University, Hsinchu, Taiwan, 1001 University Road, Hsinchu, Taiwan, 300, Republic of China; 4 Department of Photonics Engineering, Yuan Ze University, Taiwan, 135 Yuan-Tung Road, Chung-Li, Taiwan, 32003, Republic of China

**Keywords:** RF-MOMBE, InN, nanorods

## Abstract

This paper reports on high-quality InN materials prepared on a GaN template using radio-frequency metalorganic molecular beam epitaxy. We also discuss the structural and electro-optical properties of InN nanorods/films. The X-ray diffraction peaks of InN(0002) and InN(0004) were identified from their spectra, indicating that the (0001)-oriented hexagonal InN was epitaxially grown on the GaN template. Scanning electron microscopic images of the surface morphology revealed a two-dimensional growth at a rate of approximately 0.85 μm/h. Cross-sectional transmission electron microscopy images identified a sharp InN/GaN interface and a clear epitaxial orientation relationship of [0001]_InN_ // [0001]_GaN_ and ( $\bar{2}110$)_InN_ // ( $\bar{2}110$)_GaN_. The optical properties of wurtzite InN nanorods were determined according to the photoluminescence, revealing a band gap of 0.77 eV.

## Background

The narrow band gap InN has been attracting considerable attention for optoelectronic and high-speed electronic devices, thanks largely to its narrow direct band gap energy of 0.7 eV, high electron mobility, and electron saturation velocity [1-4]. Recent reports have revealed promising results in the application of InN epilayers for chemical sensors [5]. Improvements in growth techniques over the past few years have enabled the fabrication of high-quality InN epilayers through molecular beam epitaxy (MBE) [6], radio-frequency metalorganic molecular beam epitaxy (RF-MOMBE) [7], sputtering, and metalorganic chemical vapor deposition (MOCVD) [8].

Nonetheless, the epitaxial growth of InN remains a considerable challenge because a suitable lattice-matched substrate has yet to be identified, and growth conditions are greatly restricted by the low-dissociation temperature of InN. InN is usually grown on a sapphire substrate using various buffer layers, such as ZnO, GaN, AlN, and 6H-SiC [9-12]. The lattice mismatch of InN and sapphire is approximately 25%, but when GaN is used as a substrate it is only 10%, which has led to the widespread adoption of GaN as a buffer layer [13].

Previous studies on the deposition of InN on a substrate of GaN have demonstrated a substantial improvement in the nucleation of InN. Ajagunna et al*.*[14] reported that the morphology of InN depends on the N/In flux ratio used in nitrogen RF-plasma source molecular beam epitaxy (RF-MBE). Kryliouk et al*.*[15] indicated that the diameter, density, and orientation of nanorods could be controlled by temperature; the selection of substrate, and HCl/TMIn and N/In inlet molar ratios, during growth. Shubina et al*.* claimed that the narrow band gap of InN may be due to the presence of In nanoclusters, indicating that pure InN would have a large band gap [16]. The development of growth techniques, particularly in MBE, has significantly improved the quality of InN films. Nonetheless, the growth rate remains lower than that of MOCVD. Thus, the RF-MOMBE system-combining characteristics of both MBE and MOCVD techniques are suitable for the mass production of InN growth. The growth rate of RF-MOMBE is higher than the conventional MBE and is capable of producing high-quality epitaxial InN films/nanorods. Although significant progress has been made, more research is required to improve our understanding and optimization of the heteroepitaxy of InN on GaN.

However, few studies were reported about InN films growth using RF-MOMBE. MOCVD, plasma-assisted molecular beam epitaxy (PA-MBE), and metalorganic vapor phase epitaxy are the widely used techniques in InN epitaxial growth. Compared with the PA-MBE growth method, the RF-MOMBE technique generally has the advantage of a high growth rate for obtaining epitaxial nitride films [10,17]. Also, our previous study indicated that using RF-MOMBE-growth-InN-related alloys were higher than the growth rate of PA-MBE [18].

In this paper, the InN materials were grown by RF-MOMBE on the sapphire substrate using a GaN template. This paper studies how V/III flow rate and RF power influence the growth of InN films and nanorods on a GaN template using RF-MOMBE.

## Methods

We prepared InN films/nanorods on *c*-plane sapphire substrate with GaN template using MOMBE system with an RF source to activate the nitrogen. The 4-μm-thick GaN template grown by MOCVD was commercially available. A turbo molecular pump evacuated the growth chamber, reaching a base pressure of 1 × 10^−9^ Torr. The group III precursors, i.e., trimethylindium (TMIn) and trimethylaluminum, were delivered to the growth chamber by heating the metalorganic sources without carrier gases. The active nitrogen radicals were supplied by a radio-frequency plasma source. Also, the V/III flow rate was controlled by mass flow controller. During the InN growth, N_2_ flow rate were fixed at 0.6 sccm (sccm denotes cubic centimeter per minute at STP) for film and 1 sccm for rods, respectively. In our experiments, the V/III ratio was changed by adjusting the TMIn flow rate from 0.8 sccm (sample 1) to 0.4 sccm (sample 2).

The substrate was heated to 600°C for thermal cleaning, and then cooled down to 500°C for nitridation, followed by the growth of InN nanostructure at 500°C. The detailed conditions related to the deposition of InN nanostructure are listed in Table 1, and the surface morphology is identified by scanning electron microscope (SEM) images. During the deposition, the substrate and chamber temperatures were maintained using an infrared pyrometer and a thermocouple with a photoionization detector-programmable heater.

**Table 1:** Experimental parameters for the deposition of InN films/nanorods

**Sample**	**Morphology**	**Temperature (°C)**	**RF-plasma power (W)**	**V/III ratio**
1	Film	500	400	0.75
2	Rods	500	350	2.5

The surface and cross-sectional morphologies were examined using a Hitachi S-4300 field-emission scanning electron microscope (FE-SEM) (Hitachi China Ltd., Beijing, China). Structural properties were characterized by X-ray diffraction (XRD, Siemens D5000, Siemens, Cary, NC, USA) and transmission electron microscopy (TEM, Philips Tecnai 20, North Billerica, MA, USA). The Φ scan and full width at half maximum (FWHM) of the ω-scan rocking curves of InN film was measured by high-resolution X-ray diffraction (Bede D1; Bede Scientific Instruments Limited, Durham, UK). The electrical properties were measured by Hall Effect Measurement System using the van der Pauw configuration with 0.32 T magnetic field at room temperature. For Hall tests on InN, In pellets are used as ohmic metal contacts. Below, we describe metal contact fabrication using indium metal balls. The In metal pellets are 99.99% pure with a metal ball diameter of about 1 mm. The optical properties were assessed by photoluminescence at 13 K using a diode-pumped solid state laser emitting at a wavelength of 532 nm as the excitation source. The collected luminescence was directly projected into a grating spectrometer and detected with extended InGaAs detector.

## Results and discussion

Table 1 shows the growth parameters of two InN samples fabricated using RF-MOMBE. Each InN sample was grown on a 4-μm-thick GaN buffer layer. The InN nanorods were grown under N-rich condition, and the InN films were close to stoichiometry confirmed by TEM-EDX analysis.

**Figure 1:**
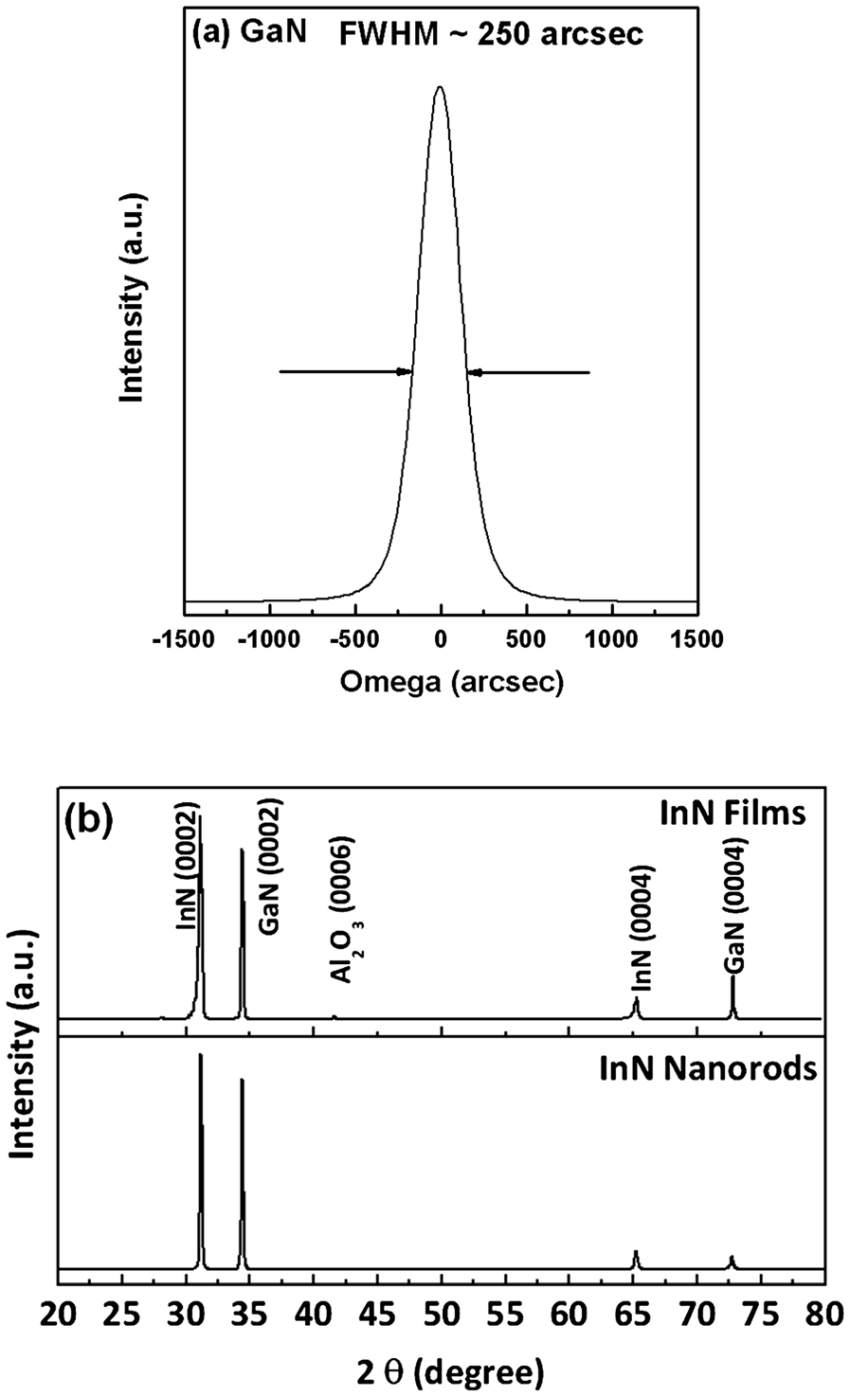
** XRD patterns of GaN and InN materials.** (**a**) ω-scan XRD profile of (0002) GaN by MOCVD; (**b**) XRD patterns of InN films/nanorods deposited on GaN template.

Figure 1a shows the XRD patterns of a high-quality GaN template and the diffraction peaks with FWHM (0002) of approximately 250 arc sec. Figure 1b exhibits the typical θ-2θ XRD profiles of InN films/nanorods grown on GaN template. The XRD patterns clearly reveal several strong diffraction peaks corresponding to the (0002) of InN, (0004) of InN, (0002) of GaN, and (0004) of GaN. These results indicate that high-quality InN films/nanorods with a hexagonal structure were preferentially oriented and grown on GaN template. In addition, the lattice parameters of InN film of the *a-* and *c*-axis were calculated as 3.55 and 5.71 Å, respectively. Previous literature reported that the *a*-axis of InN was endured and slightly strained [1]. On the contrary, our InN films had nearly relaxed on both *c*- and *a*-axis directions. The observed InN(0002) and ( $10\bar{1}2$) diffraction peaks, which are close to InN stoichiometry, suggest that the strain of InN film had nearly relaxed after growth. These values coincide with the stress-free lattice parameters of InN [19]. 

**Figure 2:**
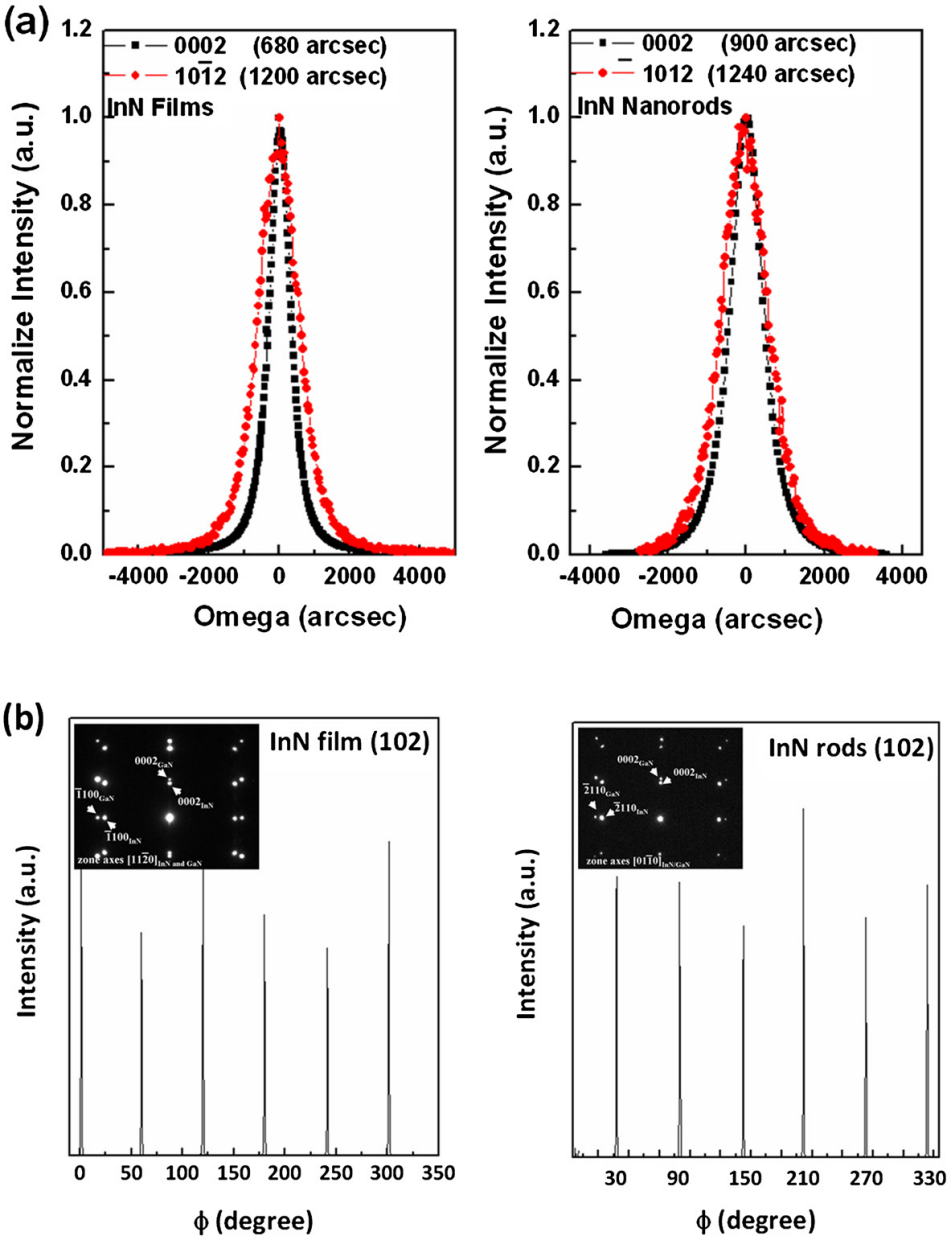
** XRC and Phi-scan patterns in XRD of InN films and InN nanorods.** (**a**) FWHM values of high-resolution X-ray diffraction InN(0002) ω-rocking curves of InN films/nanorods and (**b**) InN(1012) phi-scan of InN films/nanorods.

It has been qualitatively determined that the X-ray rocking curve for the symmetric (0002)-reflecting plane is related to the screw and mixed dislocations; whereas the X-ray rocking curve for the asymmetric ( $10\bar{1}2$)-reflecting plane is directly influenced by all threading dislocations. Figure 2a shows both InN(0002) and InN( $10\bar{1}2$) peaks for InN films and InN nanorods, respectively. For all samples, the FWHM of InN( $10\bar{1}2$) peak has a significantly greater value than that of InN(0002) peaks. InN films in particular have a minimum FWHM value of 680 and 1,200 arc sec for the symmetrical (0002) and asymmetrical ( $10\bar{1}2$) diffraction peaks which are due to the low-defect density, suggesting that the strain of InN relaxes considerably following its growth. To determine the epitaxial relationship between InN and the underlying GaN epilayers, we adopted the azimuthally Φ scan of the InN(10-12) diffraction peak. The in-plane orientations of the InN(10-12) are presented in Figure 2b. The hexagonal structure of InN(10-12) produced six equally spaced peaks. Besides, the absence of any other random peaks suggests that the InN film grains predominantly grow in the direction of [0001]. Based on the results of XRD analysis, we concluded that InN film is monocrystalline, with a normal surface on [0001] and in-plane orientation of [ $1\bar{1}20$]_InN_ // [ $1\bar{1}20$]_GaN_. These results confirm in agreement with the results of selected area electron diffraction patterns of TEM. Therefore, these results indicate that high-quality InN films/nanorods with a hexagonal structure were heteroepitaxially grown on GaN template.

**Figure 3:**
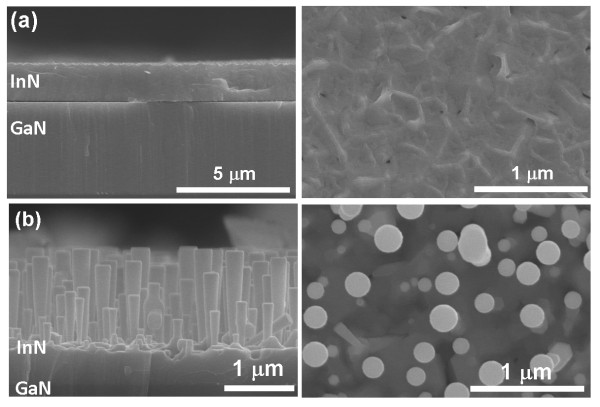
** SEM cross-sectional images of InN film/nanorods by RF-MOMBE in a 4-μm thick GaN template.** (**a**) The thickness of the film was approximately 1.7 μm with a growth rate of approximately 0.85 μm/h (**b**) Nanorods formed a cone-shaped columnar structure with separated InN columns.

Figure 3 shows a cross section and plane-view FE-SEM images of InN films/nanorods deposited on GaN template. Figure 3 (a) clearly illustrates that the thickness of the film was approximately 1.7 μm with a growth rate of approximately 0.85 μm/h under this condition. Though the growth rate is still lower than that of InN grown by MOCVD, it is faster than the conventional PA-MBE with a growth rate of about 0.6 μm/h by RF-MBE [20,21]. In addition, the surface of the film was continuous and uniform, illustrating a two-dimensional mode growth. The nanorods formed a cone-shaped columnar structure with separated InN columns, as shown in the Figure 3 (b). According to earlier literature [22], the growth condition of N-rich regime can suppress the formation of the indium droplet; and if no indium droplet appears, we speculate that the InN nanorods were grown by means of a catalytic-free growth mechanism. The average height of the columns was 1.5 μm, and the columns were aligned in the [0001] direction. Furthermore, it is noted that no droplet was observed at the end of any nanorod. Although no metallic particles were observed on the top, we cannot completely rule out the possibility of a VLS-like mechanism because In could desorb at high temperatures. The catalytic growth has been widely employed to grow 1D InN nanostructures via metals, like Au and Ni, which are used as the primary catalysts [23,24]. However, the unwanted metals might limit the potential applications of 1D InN nanostructures. Moreover, their investigation on InN nanorods growth revealed a radial random orientation from the substrates as well. In our catalytic-free growth of InN nanorods, the nanorods were on *c*-axis and well-aligned on the GaN/sapphire substrate. 

**Figure 4:**
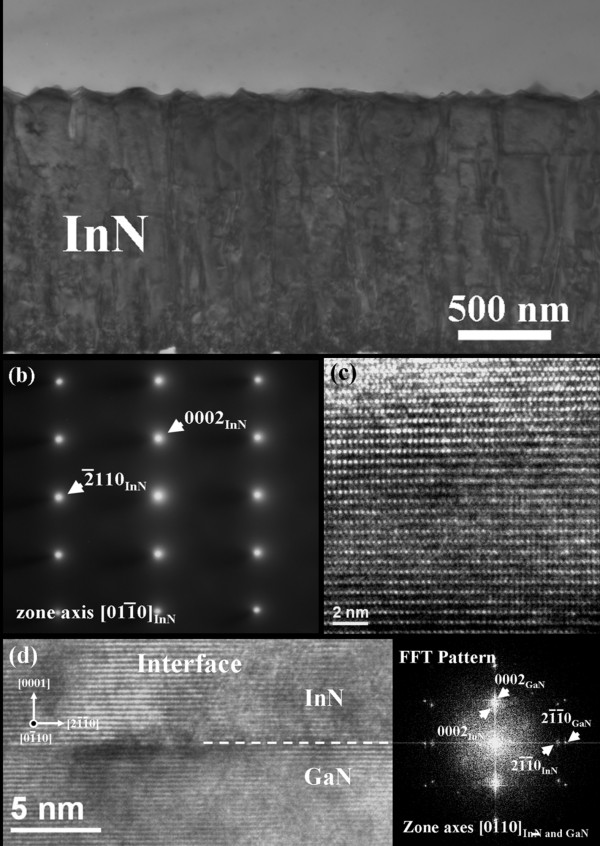
** TEM images of the cross-section of the InN/GaN.** (**a**) Cross-sectional TEM image of InN; (**b**) SAD pattern of typical InN/GaN interface; (**c**) image of 1attices of InN; and (**d**) high-resolution transmission electron microscopy of InN on GaN showing the interface and the corresponding fast-Fourier-transform pattern.

Figure 4 is a bright-field TEM image of a cross section along the zone axes of InN $01\bar{1}0$, with a corresponding selected-area diffraction (SAD) pattern of the InN film deposited on GaN template at 500°C. The thickness of the InN film was approximately 1.7 μm, clearly revealing the threading dislocations. The dislocation density is roughly estimated in the order magnitude of 2 × 10^9^ cm^−2^. Figure 4 (b) SAED pattern in the InN film and the diffraction pattern of hexagonal wurtzite structure with an incident beam direction of $01\bar{1}0$ can be clearly observed. No additional diffraction spots were observed in the pattern, implying that no interlayer existed between InN and GaN. Figure 4 (c) is a high-resolution TEM image of a cross-section of InN along the $01\bar{1}0$ direction. The (0002) lattice fringes reveal that the lattice constant of InN epilayer grown at 500°C was approximately 0.57 nm, similar to our previous TEM observations of InN on ZnO [10]. An image of the lattice in the interfacial region between InN and GaN in $01\bar{1}0$ and corresponding fast-Fourier-transform patterns are shown in Figure 4 (d). The (0002) lattice fringes reveal a sharp, smooth interface without the formation of an interlayer, indicating that no reaction occurred between them. In addition, lattice fringes with fast-Fourier-transform patterns in the inset illustrate that InN is in epitaxy with GaN. This pattern indicated that only wurtzite InN and GaN exist at the vicinity of the interface. Therefore, the coherency of InN and GaN layers across the interface is clearly visible. 

**Figure 5:**
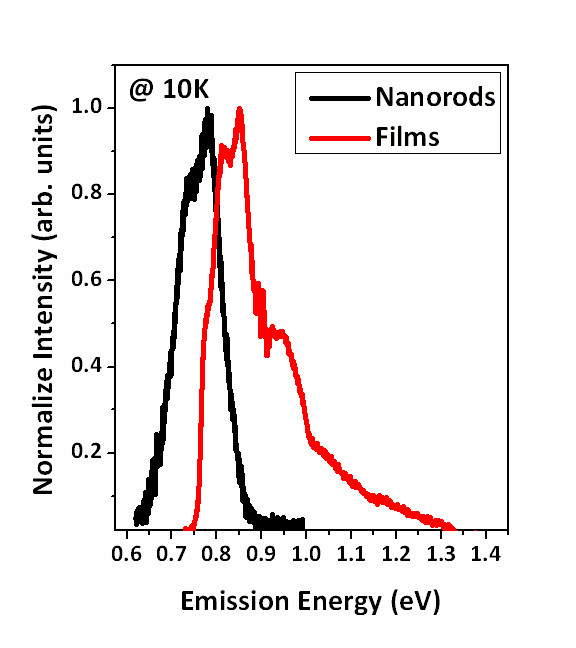
PL spectra of epitaxial InN films/nanorods grown on GaN template at 13 K.

Figure 5 shows the measurement of photoluminescence (PL) spectra at 13 K from InN films/nanorods deposited on GaN layer at 500°C. The PL spectra show that the fundamental band gap of the InN nanorods was located at 0.77 eV with FWHM of approximately 92 meV. The value measured was smaller than band gap of InN film at approximately 0.83 eV. According to the empirical function related to the FWHM of PL [25], the carrier concentration in the InN nanorods was roughly estimated to 1.1 × 10^19^ cm^−3^, which was slightly smaller than when getting from the Hall measurement. The difference may be due to the surface accumulation in the nanorods. The Hall measurements of the InN nanorods exhibited a carrier concentration of 3 × 10^19^ cm^−3^ and electron mobility of 253 cm^2^/Vs. The reduced number of defects and low background carrier concentration were the results of the high-quality InN. However, the PL emissions in InN nanorods can be attributed to the diameter of the rods, which would increase the density of crystal defects induced by the growth of mismatched lattice. Chao et al*.* characterized the PL peaks of InN nanorods as governed primarily by the quantum size effect [26]. The emission peak and FWHM of photoluminescence of our InN nanorods were smaller than those reported in [26]. The result implies that the surface accumulation in our InN nanorods has a minor influence. 

## Conclusions

In summary, this study investigated the characteristics of InN films/nanorods in epitaxial growth on the GaN template using RF-MOMBE. The structural properties shown in XRD pattern reveal the good crystallinity of InN materials grown on GaN without any metallic In phase. The TEM images indicate the sharp interface of the epitaxially grown InN/GaN. In addition, the PL spectra illustrate a band gap of 0.77 eV in the InN nanorods. These results indicate that an improvement in the quality of InN material can be achieved using heteroepitaxy on GaN template, and further investigation on the morphological revolution of InN is underway.

## Competing interests

The authors declare that they have no competing interests.

## Authors’ contributions

WCC carried out most of the experimental work including the InN materials preparation, characterization, and the RF-MOMBE growth and drafted the manuscript. WLW helped with the transmission electron microscopy experiments. JST carried out the high-resolution X-ray measurements. WTL carried out the photoluminescence measurement. FIL carried out GaN grown by MOCVD. SYK and LC were involved in the discussions of the experimental results. All authors read and approved the final manuscript.
